# Supplementary material for: Reversible Upregulation of the Senescence-Associated Beta-Galactosidase Marker Induced by Cell Detachment in Cancer Cells
Source: Cells. 2025 Oct 24;14(21):1667. doi: 10.3390/cells14211667 (PMC12609888; doi:10.3390/cells14211667)
Supplement: Supplementary file 1 [file cells-14-01667-s001.zip › cells-3796797-supplementary.pdf]

Supplementary materials

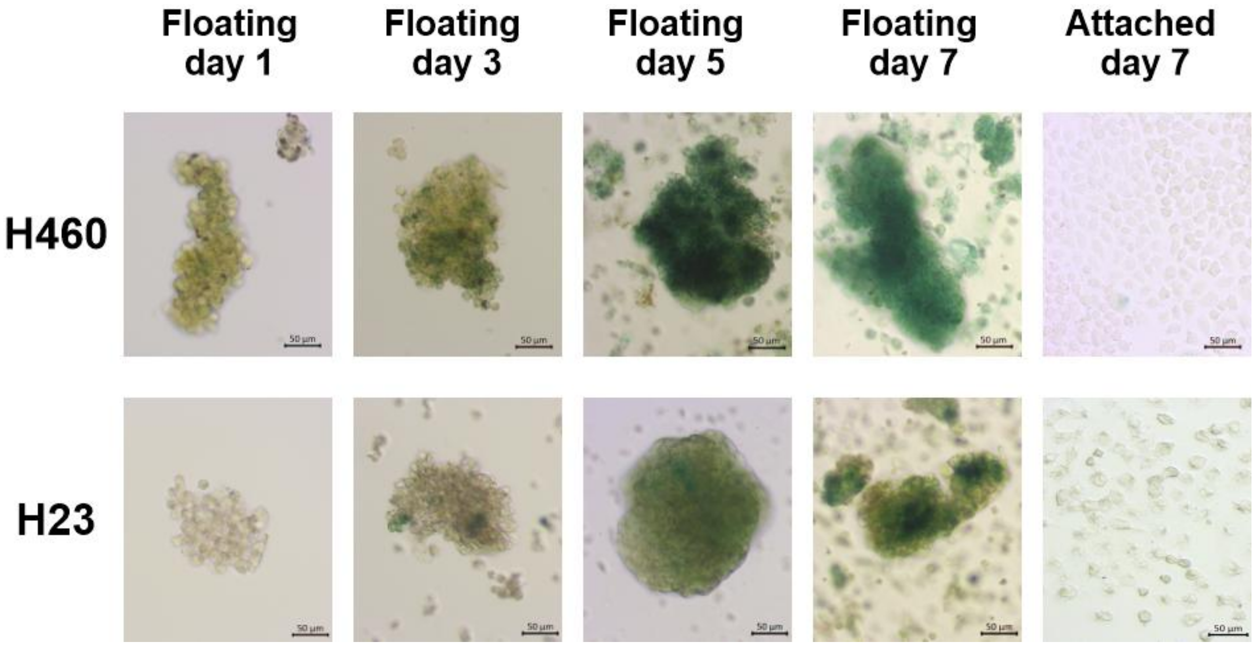

Figure S1. Progressive increase of SA-βG activity in spheroid-forming cancer cells growing under anchorage-independent conditions. H460, and H23 cells grown for 7 days under AICs (floating) were collected at the indicated times, fixed and stained for SA-βG activity. Images were taken at 5x magnification. H460 and H23 cells grown for 7 days under ADCs (attached) were used as a control.

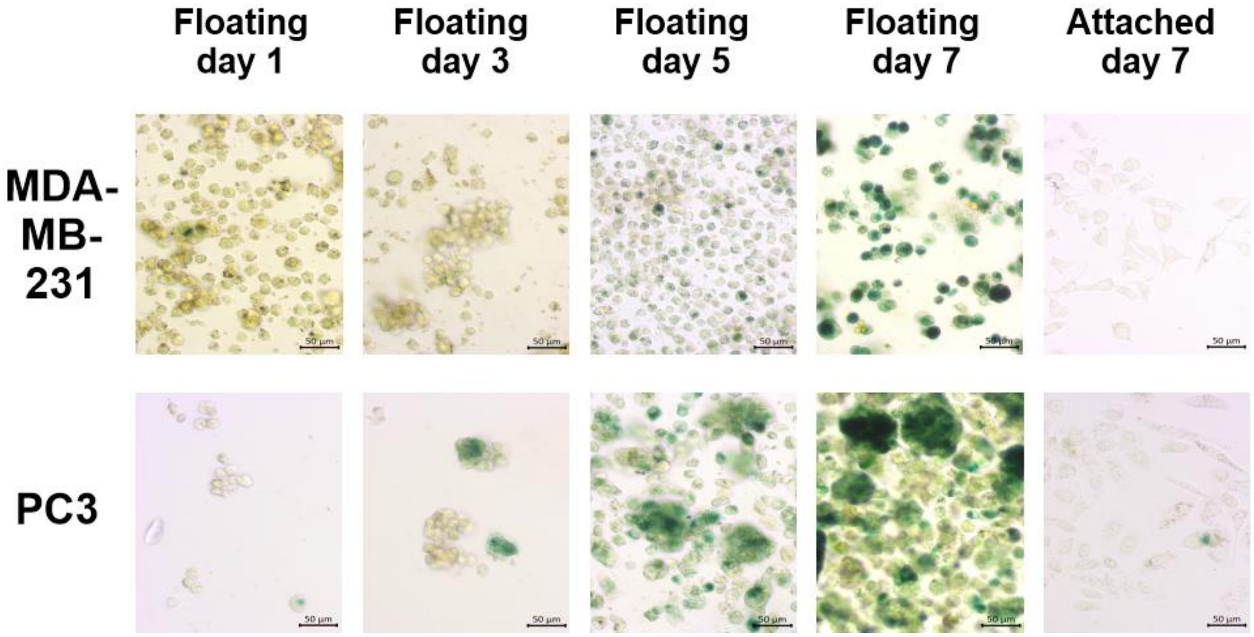

Figure S2. Progressive increase of SA-βG activity in non-spheroids-forming cancer cells growing under anchorage-independent conditions. MDA-MB-231 and PC3 cells grown for 7 days under AICs (floating) were collected at the indicated times, fixed and stained for SA-βG activity. Images were taken at 5x magnification. MDA-MB-231 and PC3 cells grown for 7 days under ADCs (attached) were used as a control.

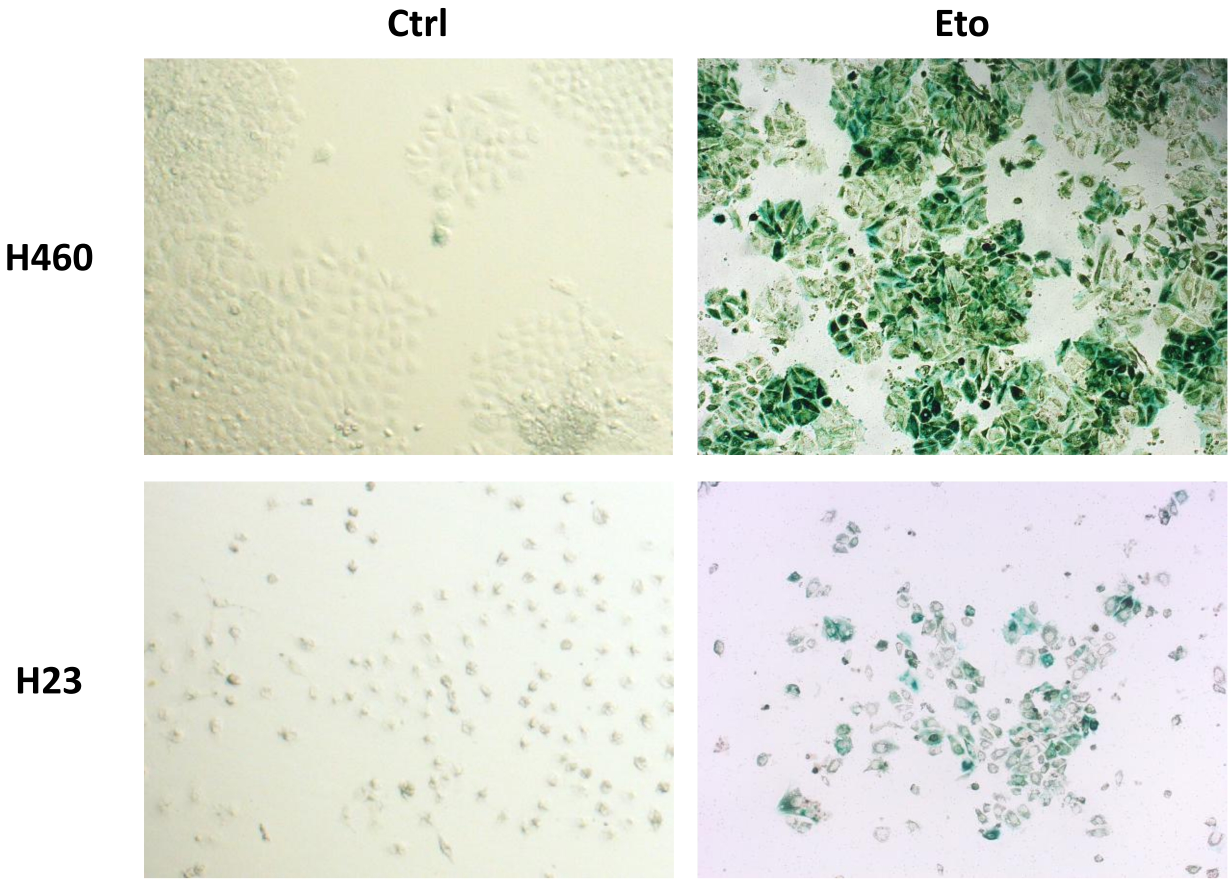

Figure S3. Eto induced a Q/S state in attached H460 and H23 cells. Cells grown under ADCs for 6 days in the presence of 1.25 μM Eto. Samples were stained for SA-βG activity. Images were taken at 5x magnification.

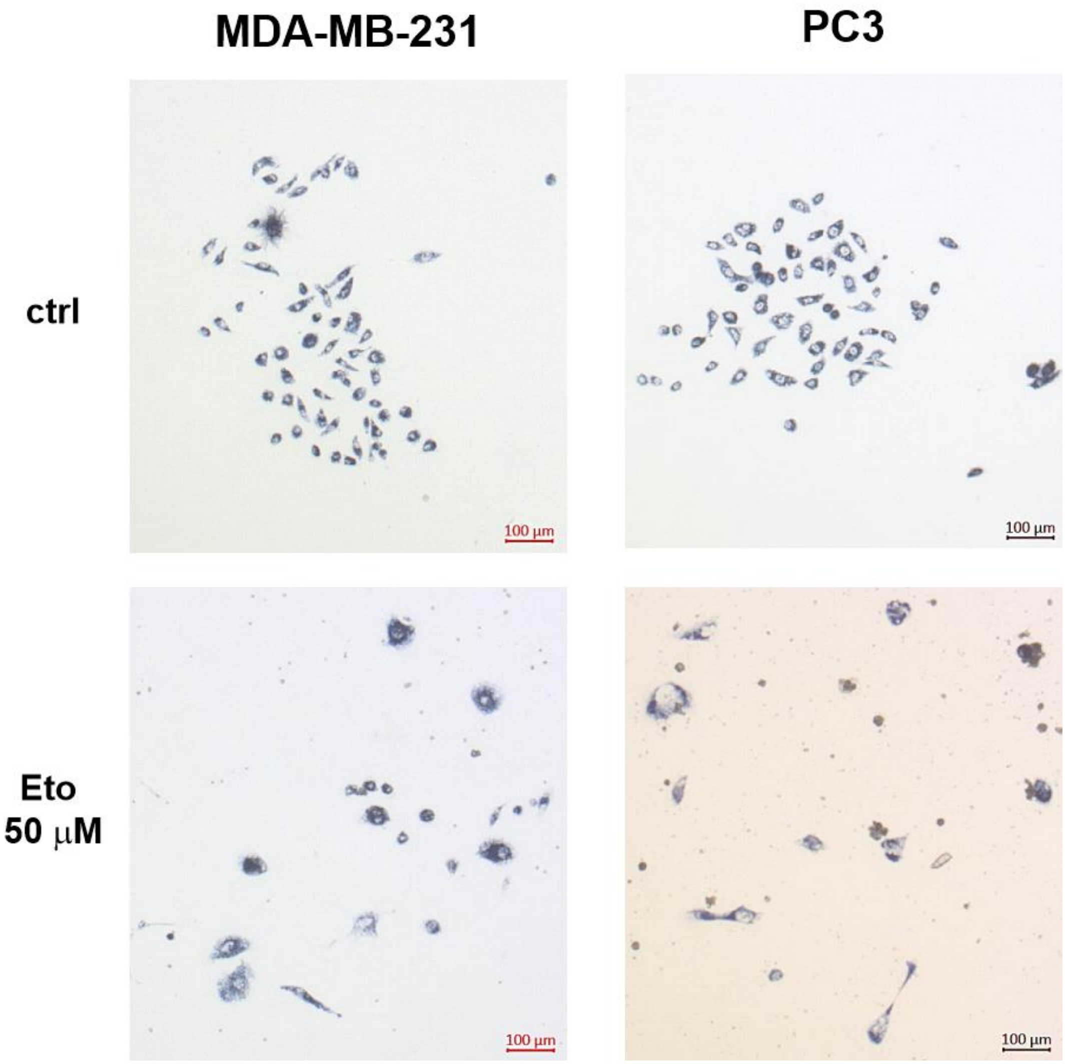

Figure S4. SA-βG⁺ cells remain metabolically active. MDA-MB-231 and PC3 cells grown under AICs for seven days in the absence or presence of Eto were allowed to reattach for 7 days and then incubated with MTT.

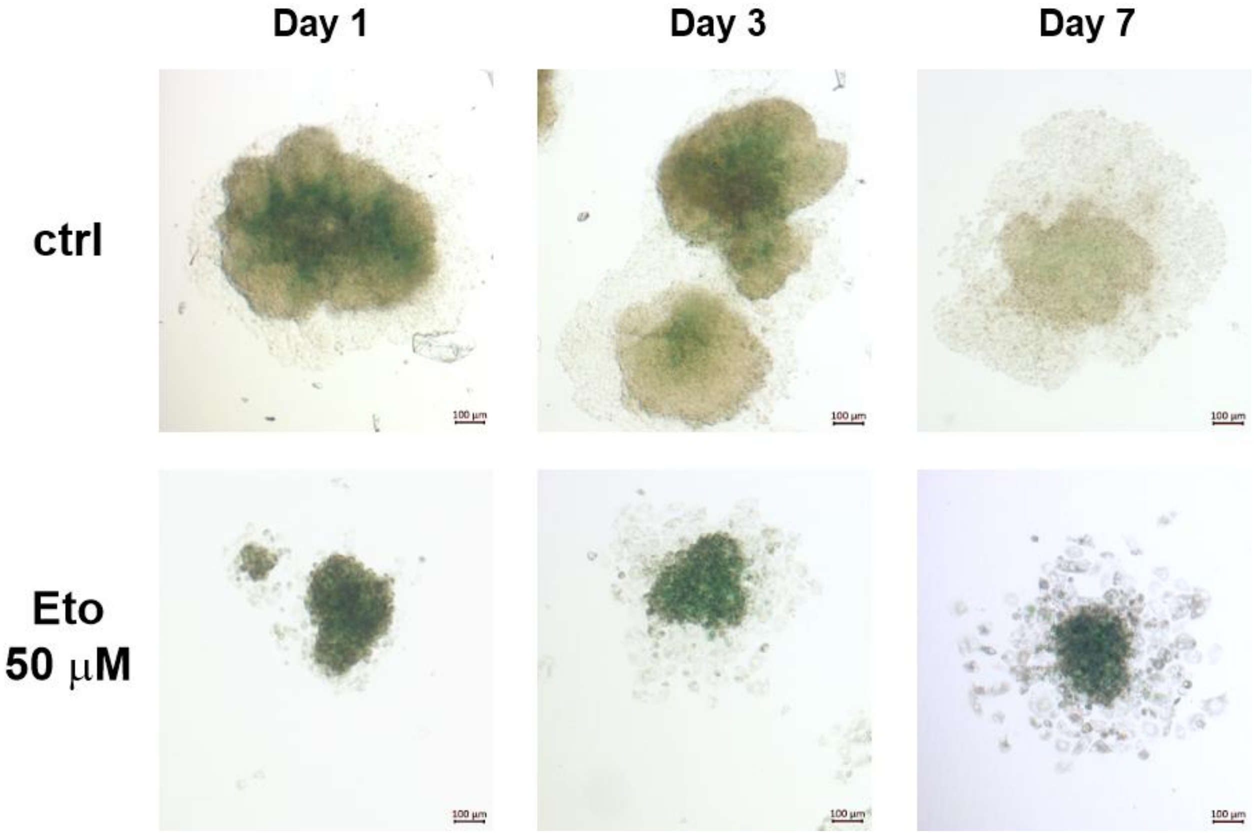

Figure S5. Rapid decrease of SA-βG activity upon cell-plate contact. H460 cells grown under AICs for seven days in the absence or presence of Eto were allowed to reattach. Samples were collected at the indicated time and stained for SA-βG activity.

Table S1. The topmost differentially expressed Kegg carbohydrate metabolism / Leloir pathway genes.

Gene Symbol	Pathway	Expression	LogFC	P-value (Survival)
ALDH1L1	Dicarboxylate metabolism	DOWN	-4.84	9.9e-177
ATP1A2	Ascorbate metabolism	DOWN	-5.1	6.96e-172
GPD1	Gluconeogenesis pathway	DOWN	-5.9	4.01e-169
RBP4	Gluconeogenesis pathway	DOWN	-5.37	5.09e-163
LEPR	Gluconeogenesis pathway	DOWN	-3.06	9.29e-163
ASPA	Dicarboxylate metabolism	DOWN	-3.58	1.53e-159
ACACB	Pentose phosphate pathway	DOWN	-3.22	1.6e-145
KL	Pentose glucuronate interconversions	DOWN	-2.86	1.79e-136
PCK1	Gluconeogenesis pathway	DOWN	-5.81	3.73e-133
PFKFB1	Glycolytic processes	DOWN	-3.01	5.66e-114
ACSS2	Propanoate metabolism	DOWN	-1.4	6.78e-97
ACO1	TCA cycle pathway	DOWN	-1.46	3.12e-83
SLC35A2	Galactose metabolism	UP	1.31	3.3e-81
HTR2A	Glycolytic processes	DOWN	-3.08	1.04e-76
ALDOC	Glycolytic processes	DOWN	-2.3	9.09e-71
SDS	Gluconeogenesis pathway	UP	2.74	4.37e-67
KAT2B	Gluconeogenesis pathway	DOWN	-1.34	3.97e-66
ALDH18A1	Dicarboxylate metabolism	UP	1.03	4.01e-66
ATF3	Gluconeogenesis pathway	DOWN	-2.3	5.47e-64
UGP2	NANA	DOWN	-1.03	2.15e-62
ADHFE1	Dicarboxylate metabolism	DOWN	-1.85	1.99e-61
PPARA	Gluconeogenesis pathway	DOWN	-1.47	6.03e-59
MTHFD2	Dicarboxylate metabolism	UP	1.4	6.04e-57
PER2	Gluconeogenesis pathway	DOWN	-1.12	4.1e-56
PFKFB4	Fructose metabolism	UP	1.34	3.49e-53
PFKFB3	Glycolytic processes	DOWN	-1.65	1.62e-52
TAT	Dicarboxylate metabolism	DOWN	-4.26	8.87e-51
GLB1L3	Galactose metabolism	DOWN	-2.72	1.07e-50
PPARGC1A	Gluconeogenesis pathway	DOWN	-2.43	1.83e-50
ATIC	Dicarboxylate	UP	0.872	2.09e-46

	metabolism			
SLC16A3	Pyruvate metabolism	UP	2.16	2.2e-45
GALE	Galactose metabolism	UP	1.53	3.35e-45
PKM	Glycolytic processes	UP	1.01	4.39e-45
SHMT1	Dicarboxylate metabolism	DOWN	-0.93	5.71e-45
HOGA1	Pyruvate metabolism	DOWN	-2.39	4.3e-44
H6PD	Pentose phosphate pathway	DOWN	-0.796	3.05e-40
ADSS2	Dicarboxylate metabolism	UP	0.846	1.99e-38
SUCLA2	TCA cycle pathway	DOWN	-0.724	2.05e-38
IDH2	TCA cycle pathway	UP	1.29	2.28e-38
HAAO	Dicarboxylate metabolism	DOWN	-1.32	4.95e-38
ACADS	Butanoate metabolism	DOWN	-1.32	3.11e-37
PGM1	Galactose metabolism	DOWN	-0.979	3.12e-34
PM20D2	Dicarboxylate metabolism	DOWN	-1.61	1.02e-33
MAN2A2	Mannose metabolism	DOWN	-0.813	2.96e-33
GMPPB	Mannose metabolism	UP	0.822	3.07e-33
SLC2A1	Ascorbate metabolism	UP	1.35	3.25e-33
GLB1	Galactose metabolism	UP	0.677	1.75e-32
FAHD1	Pyruvate metabolism	UP	0.817	2.44e-32
FOLR1	Dicarboxylate metabolism	DOWN	-3.01	5.94e-32
AGXT	Glyoxylate metabolism	DOWN	-2.27	1.49e-31
PMM2	Mannose metabolism	UP	0.909	2.02e-31
SORD	Fructose metabolism	UP	1.62	2.53e-31
TPI1	Glycolytic processes	UP	0.919	3.54e-31
FOXK2	Glycolytic processes	UP	0.74	7.05e-31
VDAC1	Pyruvate metabolism	UP	0.668	1.1e-30
PC	Gluconeogenesis pathway	DOWN	-1.08	1.86e-30
DHTKD1	Glycolytic processes	UP	0.947	2.04e-30
ME3	Pyruvate metabolism	DOWN	-1.13	7.42e-30
SLC23A2	Ascorbate metabolism	DOWN	-0.728	6.29e-29
SLC19A1	Dicarboxylate metabolism	UP	1.01	2.48e-28
SDHC	TCA cycle pathway	UP	0.657	5.9e-28
GCK	Glycolytic processes	DOWN	-1.4	2.19e-27
HK1	Glycolytic processes	UP	0.61	4.2e-27
GAPDH	Glycolytic processes	UP	0.931	1.85e-26
WDR5	Gluconeogenesis pathway	UP	0.529	1.02e-24
ALDOB	Glycolytic processes	DOWN	-1.33	2.29e-24
PGK1	Glycolytic processes	UP	0.773	3.48e-24
CLSTN3	Ascorbate metabolism	UP	0.827	6.89e-24
GLYCTK	Fructose metabolism	DOWN	-0.777	9.26e-24
SLC25A10	Gluconeogenesis pathway	UP	1.14	9.5e-24
GALT	Galactose metabolism	DOWN	-0.715	2.36e-23

ALDOA	Glycolytic processes	UP	1.08	6.41e-23
SUCLG1	TCA cycle pathway	DOWN	-0.453	8.64e-23
AKR1A1	Pentose glucuronate interconversions	UP	0.603	1.75e-22
GOT2	Dicarboxylate metabolism	UP	0.732	3.2e-22
ACOT8	Dicarboxylate metabolism	UP	0.558	3.66e-22
LDHB	Pyruvate metabolism	DOWN	-1.29	4.17e-22
MAN2C1	Mannose metabolism	DOWN	-0.569	1.1e-21
FH	TCA cycle pathway	UP	0.619	1.15e-21
PRPS2	Pentose phosphate pathway	UP	0.739	2.04e-21
DLST	TCA cycle pathway	DOWN	-0.416	5.95e-21
GOT1	Gluconeogenesis pathway	UP	0.612	1.85e-20
ME1	Pyruvate metabolism	DOWN	-1.31	8.63e-20
NIT2	Dicarboxylate metabolism	UP	0.443	5.33e-19
CRTC2	Gluconeogenesis pathway	UP	0.513	7.11e-19
GAA	Sucrose metabolism	UP	0.701	1.32e-18
ADPGK	Glycolytic processes	UP	0.342	3.24e-18
IER3	Glycolytic processes	UP	1.28	5.98e-18
UGT2B7	Pentose glucuronate interconversions	DOWN	-2.08	2.36e-17
MDH2	Gluconeogenesis pathway	UP	0.545	2.84e-17
DGLUCY	Dicarboxylate metabolism	DOWN	-0.608	8.15e-17
TKFC	Fructose metabolism	UP	0.627	1.03e-16
PGLS	Pentose phosphate pathway	UP	0.608	1.32e-16
PFKL	Glycolytic processes	UP	0.596	3.1e-16
FAHD2A	Dicarboxylate metabolism	DOWN	-0.492	3.89e-16
ALDH4A1	Dicarboxylate metabolism	DOWN	-0.805	9.29e-16
UGT2B11	Pentose glucuronate interconversions	DOWN	-3.03	9.68e-16
GALK1	Galactose metabolism	UP	0.801	1.08e-15
PCK2	Gluconeogenesis pathway	UP	0.684	1.17e-15
CHST1	Galactose metabolism	UP	1.53	2.68e-15
SDHAF2	TCA cycle pathway	UP	0.535	2.95e-15
UGT1A7	Pentose glucuronate interconversions	DOWN	-1.41	3.29e-15
SDHD	TCA cycle pathway	DOWN	-0.479	5.07e-15
MAN2B1	Mannose metabolism	UP	0.518	7.5e-15
UGT2B28	Pentose glucuronate interconversions	DOWN	-2.32	1.07e-14
GPI	Glycolytic processes	UP	0.572	1.44e-13
G6PD	Pentose phosphate	UP	0.692	1.56e-13

	pathway			
RBKS	Pentose phosphate pathway	UP	0.589	1.62e-13
G6PC3	Gluconeogenesis pathway	UP	0.633	1.71e-13
MPC2	Pyruvate metabolism	UP	0.624	4.43e-13
B4GALT1	Galactose metabolism	UP	0.575	1.8e-12
PGAM1	Glycolytic processes	UP	0.41	1.81e-12
XYLB	Pentose glucuronate interconversions	UP	0.562	1.92e-12
ACLY	Dicarboxylate metabolism	UP	0.485	2.28e-12
ENO2	Glycolytic processes	UP	1.02	2.65e-12
ERO1A	Ascorbate metabolism	UP	0.573	3.9e-12
ACOD1	C5 branched dibasic acid metabolism	UP	1.36	4.3e-12
LDHA	Glycolytic processes	UP	0.522	5.9e-12
ASS1	Dicarboxylate metabolism	DOWN	-1.11	1.28e-11
DDO	Dicarboxylate metabolism	DOWN	-0.815	1.3e-11
SLC23A1	Ascorbate metabolism	UP	1.1	2.25e-11
KMO	Dicarboxylate metabolism	UP	1.33	2.76e-11
LDHAL6A	Pyruvate metabolism	DOWN	-0.939	3.65e-11
G6PC2	Gluconeogenesis pathway	DOWN	-1.1	4.32e-11
ASL	Dicarboxylate metabolism	UP	0.501	4.94e-11
L2HGDH	Dicarboxylate metabolism	UP	0.454	2.14e-10
BPGM	Glycolytic processes	UP	0.351	2.38e-10
GSTO1	Ascorbate metabolism	UP	0.394	2.58e-10
TALDO1	Pentose phosphate pathway	UP	0.451	4.09e-10
GUSB	Pentose glucuronate interconversions	UP	0.475	5.02e-10
RPE	Pentose phosphate pathway	UP	0.318	5.25e-10
UGT2B4	Pentose glucuronate interconversions	DOWN	-1.67	6.31e-10
AKR1B1	Fructose metabolism	DOWN	-0.524	7.56e-10
LDHAL6B	Pyruvate metabolism	DOWN	-0.8	1.7e-09
QPRT	Dicarboxylate metabolism	UP	1.05	1.91e-09
SLC2A3	Ascorbate metabolism	DOWN	-0.618	2.42e-09
ACOT4	Dicarboxylate metabolism	UP	0.764	5.45e-09
SLC37A4	Gluconeogenesis pathway	UP	0.351	6.61e-09
DCXR	Pentose glucuronate interconversions	UP	0.622	8.16e-09
PGK2	Glycolytic processes	UP	0.986	9.82e-09
GPT2	Dicarboxylate	DOWN	-0.66	1.89e-08

	metabolism			
MGAT4A	Glyoxylate metabolism	UP	0.607	2.65e-08
MDH1	TCA cycle pathway	DOWN	-0.3	1.22e-07
SDHAF4	TCA cycle pathway	DOWN	-0.32	1.71e-07
LIPA	Glycolytic processes	DOWN	-0.377	2.84e-07
IDH3G	TCA cycle pathway	UP	0.311	4.54e-07
SLC25A13	Gluconeogenesis pathway	UP	0.338	5.04e-07
CRYL1	Pentose glucuronate interconversions	DOWN	-0.376	5.47e-07
IDH1	TCA cycle pathway	DOWN	-0.427	5.66e-07
GCLM	Dicarboxylate metabolism	DOWN	-0.342	9.25e-07
HK3	Glycolytic processes	UP	0.812	9.71e-07
UGDH	Pentose glucuronate interconversions	UP	0.591	9.78e-07
SERPINA12	Gluconeogenesis pathway	UP	1.37	9.89e-07
BCL2L13	Glycolytic processes	DOWN	-0.249	1.41e-06
GCLC	Ascorbate metabolism	DOWN	-0.329	1.52e-06
FPGS	Dicarboxylate metabolism	UP	0.314	1.59e-06
MTHFD1	Dicarboxylate metabolism	DOWN	-0.274	3.83e-06
PDHX	Pyruvate metabolism	UP	0.277	6.16e-06
UCP2	Glycolytic processes	UP	0.546	6.71e-06
GPD2	Gluconeogenesis pathway	UP	0.365	1.81e-05
AADAT	Dicarboxylate metabolism	DOWN	-0.585	1.94e-05
ALDH5A1	Dicarboxylate metabolism	DOWN	-0.432	2.06e-05
RPIA	Pentose phosphate pathway	UP	0.228	2.24e-05
ADSS1	Dicarboxylate metabolism	DOWN	-0.443	2.83e-05
CSKMT	TCA cycle pathway	UP	0.347	3.65e-05
BLOC1S6	Dicarboxylate metabolism	DOWN	-0.187	5.01e-05
KAT2A	Gluconeogenesis pathway	UP	0.314	6.58e-05
GAD1	Dicarboxylate metabolism	UP	0.971	6.94e-05
FBP1	Gluconeogenesis pathway	UP	0.696	7.65e-05
PHYH	Dicarboxylate metabolism	DOWN	-0.321	0.000117
CS	TCA cycle pathway	DOWN	-0.208	0.000119
UGT2B15	Pentose glucuronate interconversions	DOWN	-1.15	0.00012
NAGS	Dicarboxylate metabolism	UP	0.55	0.000125
ME2	Pyruvate metabolism	UP	0.284	0.000252

SDHA	TCA cycle pathway	UP	0.227	0.000427
COL6A1	Glycolytic processes	UP	0.428	0.000489
DLD	Pyruvate metabolism	DOWN	-0.194	0.000495
SRR	Pyruvate metabolism	DOWN	-0.219	0.000513
ENO3	Glycolytic processes	UP	0.36	0.000528
MFSD8	Glycolytic processes	DOWN	-0.167	0.000532
MTHFD1L	Dicarboxylate metabolism	UP	0.387	0.000741
SDHAF3	Dicarboxylate metabolism	DOWN	-0.374	0.000868
GLS	Dicarboxylate metabolism	DOWN	-0.278	0.000965
SLC46A1	Dicarboxylate metabolism	DOWN	-0.31	0.000989
PGAM4	Glycolytic processes	UP	0.35	0.00132
SLC25A11	Gluconeogenesis pathway	UP	0.197	0.00132
FOXK1	Glycolytic processes	DOWN	-0.228	0.00137
PFKFB2	Glycolytic processes	UP	0.341	0.00152
TIGAR	Glycolytic processes	UP	0.244	0.00165
PDHB	TCA cycle pathway	UP	0.171	0.00193
ACSS1	Propanoate metabolism	UP	0.345	0.002
DHFR	Dicarboxylate metabolism	UP	0.265	0.00219
SLC39A14	Gluconeogenesis pathway	DOWN	-0.239	0.0024
PPP4R3A	Gluconeogenesis pathway	UP	0.134	0.0026
IDH3A	TCA cycle pathway	DOWN	-0.216	0.00261
IDO1	Dicarboxylate metabolism	UP	0.66	0.00524
GALK2	Galactose metabolism	DOWN	-0.17	0.00533
FBP2	Gluconeogenesis pathway	UP	0.522	0.00539
GLUD1	Dicarboxylate metabolism	DOWN	-0.171	0.0066
IDH3B	TCA cycle pathway	UP	0.152	0.00714
ACSM1	Butanoate metabolism	DOWN	-0.508	0.00767
KYAT3	Dicarboxylate metabolism	DOWN	-0.16	0.00819
DHFR2	Dicarboxylate metabolism	DOWN	-0.157	0.0105
GRHPR	Glyoxylate metabolism	UP	0.134	0.011
MDH1B	TCA cycle pathway	UP	0.325	0.0116
SUCLG2	TCA cycle pathway	DOWN	-0.159	0.0116
MTHFS	Dicarboxylate metabolism	UP	0.207	0.0126
GGT1	Dicarboxylate metabolism	UP	0.467	0.0139
SELENON	Ascorbate metabolism	UP	0.133	0.0148
ENO1	Glycolytic processes	UP	0.215	0.0158

MTRR	Dicarboxylate metabolism	UP	0.16	0.019
SDHB	TCA cycle pathway	UP	0.123	0.0197
AMDHD1	Dicarboxylate metabolism	DOWN	-0.387	0.0224
MTHFD2L	Dicarboxylate metabolism	DOWN	-0.155	0.0273
AASDHPPT	Dicarboxylate metabolism	UP	0.132	0.0291
UGT2B10	Pentose glucuronate interconversions	UP	0.484	0.0308
TKT	Pentose phosphate pathway	DOWN	-0.178	0.0379
AGXT2	Glyoxylate metabolism	UP	0.414	0.047
GSTO2	Ascorbate metabolism	UP	0.281	0.0471
NDP	TCA cycle pathway	DOWN	-0.556	0.048
GLUD2	Dicarboxylate metabolism	DOWN	-0.218	0.0487
